# Sex Determination from Fragmented and Degenerated DNA by Amplified Product-Length Polymorphism Bidirectional SNP Analysis of Amelogenin and SRY Genes

**DOI:** 10.1371/journal.pone.0169348

**Published:** 2017-01-04

**Authors:** Kotoka Masuyama, Hideki Shojo, Hiroaki Nakanishi, Shota Inokuchi, Noboru Adachi

**Affiliations:** 1 Department of Legal Medicine, Interdisciplinary Graduate School of Medicine and Engineering, University of Yamanashi, Chuo, Yamanashi, Japan; 2 Department of Forensic Medicine, Juntendo University School of Medicine, Bunkyo-ku, Tokyo, Japan; Seoul National University College of Medicine, REPUBLIC OF KOREA

## Abstract

Sex determination is important in archeology and anthropology for the study of past societies, cultures, and human activities. Sex determination is also one of the most important components of individual identification in criminal investigations. We developed a new method of sex determination by detecting a single-nucleotide polymorphism in the amelogenin gene using amplified product-length polymorphisms in combination with sex-determining region Y analysis. We particularly focused on the most common types of *postmortem* DNA damage in ancient and forensic samples: fragmentation and nucleotide modification resulting from deamination. Amplicon size was designed to be less than 60 bp to make the method more useful for analyzing degraded DNA samples. All DNA samples collected from eight Japanese individuals (four male, four female) were evaluated correctly using our method. The detection limit for accurate sex determination was determined to be 20 pg of DNA. We compared our new method with commercial short tandem repeat analysis kits using DNA samples artificially fragmented by ultraviolet irradiation. Our novel method was the most robust for highly fragmented DNA samples. To deal with allelic dropout resulting from deamination, we adopted “bidirectional analysis,” which analyzed samples from both sense and antisense strands. This new method was applied to 14 Jomon individuals (3500-year-old bone samples) whose sex had been identified morphologically. We could correctly identify the sex of 11 out of 14 individuals. These results show that our method is reliable for the sex determination of highly degenerated samples.

## Introduction

Sex determination of ancient humans is important in the study of ancient cultures, societies, archeological histories, and genealogies. In criminal investigations, sex determination is also essential for individual identification. Because morphological sex determination is not applicable to forensic and archeological samples that lack morphological diagnostic characteristics, such as fragmentary bones and body fluid samples, sex determination based on DNA analysis is critical.

Several PCR-based methods for sex determination have been established by analyzing Y-specific target sequences on the Y chromosome, such as Y-specific repetitive sequences from DYS14 and DYZ3 [1]. Sex-specific genes, such as sex-determining region Y (SRY), have also been used as genetic markers [2–4]. Pfitzinger et al. have introduced a coamplification method using X-specific repetitive sequences from DXS424 and Y-specific repetitive sequences from DYZ1 [5] for DNA-based sex determination analysis. Additional DNA-based sex typing methods have been proposed that target X-Y homologous genes with insertion deletions, such as centromeric alphoid repeats [6, 7], zinc-finger protein genes (ZF) [8, 9], and amelogenin genes (AMEL) [10–12].

Among them, the amelogenin sex test is the most widely used in forensic practice and anthropological studies. Several methods based on detection of the amelogenin gene have been reported [10–12] and the most commonly used amelogenin primer sets were developed by Sullivan et al. [11]. These primers target a 6-bp insertion/deletion within an intron of the amelogenin gene on the X and Y chromosomes (AMELX and AMELY) and produce 106-bp and 112-bp amplicons for the X and Y chromosomes, respectively [11]. These primer sets are incorporated into commercially available STR kits, such as Promega’s PowerPlex^®^ 16 System and Applied Biosystem’s AmpFlSTR^®^ Profiler Plus^®^. However, with these conventional methods, it is difficult to determine sex from highly fragmented/degenerated DNA samples or from small sample quantities because DNA testing depends heavily on the quality and quantity of DNA samples.

The average fragment length of ancient DNA is between 100 and 500 bp [13, 14]. Ancient DNA samples are frequently broken down into small fragments and are small in quantity [14] because DNA is easily degraded by environmental factors and microbial attack at archeological sites. Fragmented forensic samples also make it difficult to investigate criminal cases. One of the major mechanisms of DNA fragmentation is depurination or depyrimidination caused by the hydrolysis of N-glycosidic bonds. Depurination/depyrimidination breaks N-glycoside bonds between sugars and nitrogenous bases [15], resulting in the release of purine (adenine: A, guanine: G) and pyrimidine bases (cytosine: C, thymine: T) from nucleotides, which in turn produces a baseless site, also known as an AP site (apurinic/apyrimidinic site) [16, 17]. Baseless sites make DNA molecules unstable and induce DNA strand separation by a β-elimination process [15]. Fragmented and low-quantity template DNA samples can cause allelic dropout, which may result in the incorrect identification of heterozygotes as homozygotes. Therefore, the shortening of PCR products is essential for DNA analyses with highly fragmented samples. Furthermore, SNP analysis is better than conventional methods because SNP primers that generate a short PCR amplicon can easily be designed.

However, DNA can be modified by spontaneous chemical reactions, such as hydrolytic reactions. Hydrolysis not only causes short products, but also induces *postmortem* DNA modifications, called miscoding lesions. Deamination, the most common type of DNA modification in highly degraded samples, removes an amino group (NH_2_) from a molecule [14]. Deamination leads to base substitutions, and it modifies A to hypoxanthine (H), G to xanthine (X), and C to uracil (U) [18, 19, 20]. In ancient samples, base substitution due to deamination is a significant problem [14], and substituted bases can cause incorrect results due to their ability to form unexpected base pairings. H can base pair with all bases, A, G, C, and T [19]. The thermodynamic stability of H base pairs is as follows: H:C > H:A > H:T ≈ H:G [18], and H residues mainly combine with C residues in PCR [21]. X has the capacity to pair with C via two hydrogen bonds just as G does and so is not miscoding [20], whereas U is able to base pair with a noncognate base, A [14]. In addition, the predominant type of nucleotide misincorporation in highly degraded ancient samples resulting from deamination is type 2 transitions (C→T and G→A) [21–24]. C bases are substantially more susceptible to hydrolytic deamination than other bases [21, 23] and C→T transitions are mainly generated by the deamination of C bases [24]. In the SNP analysis of highly degenerated samples, template DNA modifications by deamination may cause the mismatch of complementary SNP primers and/or allelic dropout, leading to false and/or negative results. Because *postmortem* DNA damage contributes to shortened amplicons as well as significant changes in DNA sequences, a strategy for SNP primer design that encompasses highly degenerated DNA samples is critical.

PCR-APLP is an SNP typing method [25–33] based on PCR that uses allele-specific primers containing SNP sites at the 3′-terminus of each primer. To use this method, at least two allele-specific primers and one “counter primer,” which serves as a common sense or antisense strand of the allele-specific primers, are required. The allele-specific primers have SNP sites at the 3′-termini, and one of these primers should have a few noncomplementary flaps at the 5ʹ-terminus to detect SNPs by determining the difference of amplicon length by PCR and subsequent electrophoresis. We previously reported a more reliable SNP analysis method based on PCR-APLP using a 5ʹ-inosine flap primer, which improves the competitiveness of allele-specific primers [33]. In this study, we introduce a novel PCR-APLP sex determination method based on the detection of an SNP in exon 2 of the AMYL gene. We present an SNP primer design strategy for highly fragmented and degenerated DNA samples. In particular, to examine the influence of *postmortem* DNA modifications in ancient samples, we detect a C↔G transversion polymorphism from both sense and antisense strands and confirm allelic dropout in degenerated DNA using the SNP primers for deamination analysis. Furthermore, the SRY gene, as an alternative Y-specific marker, is coamplified with the AMEL gene because large-region deletions in the Y chromosome are frequently observed and responsible for AMELY allelic dropout, resulting in incorrect conclusions regarding the sex associated with the sample [34–50]. Thus, this novel sense–antisense AMEL PCR-APLP assay with SRY analysis incorporates “bidirectional analysis” to examine samples from both sense and antisense strands to enable the analysis of massively degraded DNA.

## Materials and Methods

### Sample preparation

To obtain modern-day DNA samples, intraoral epithelial cells were collected from eight unrelated Japanese individuals (four male, four female). Before the cells were collected, volunteers were informed in writing that their DNA would be anonymized and that it would be used only for sex determination. Written consent to use their DNA was then obtained from each volunteer in the study. Both the consent procedure and the written forms were approved by the ethics committee of the Faculty of Medicine, University of Yamanashi (approval number 1299).

We collected samples using Forensic Swabs (Salstedt, Nümbrecht, Germany), and extracted DNA from oral mucosa cells using a Monofas^®^ Genomic DNA Extraction Kit VIII (GL Sciences, Tokyo, Japan), in accordance with the manufacturers’ recommendations. The quantity and purity of the extracted DNA were evaluated by optical density (OD)_260_ and OD_260/280_ measurements using a spectrophotometer (NanoDrop 1000; Thermo Fisher Scientific Inc., Waltham, MA, USA). Male and female human genomic DNAs of known concentrations (Promega, Madison, WI, USA) were used as standards.

### Primer design and testing

To examine samples from both sense and antisense strands of amelogenin genes, two primer sets were used (sets of sense and antisense primers). Amelogenin genes comprise seven exons and eight introns, the coding exons 2–6 being highly conserved [51]. Therefore, a sex test based on the analysis of these coding regions is expected to be more reliable than one based on intron analysis. Exons 2 and 6 of amelogenin genes have C↔G transversion polymorphisms. Exon 6 has multiple SNPs in a small region. A counter primer in APLP requires a primer binding site that does not have any mutations; therefore, we detected a C↔G transversion polymorphism in exon 2 of the amelogenin gene.

### Sense primer sets

**Fig 1:**
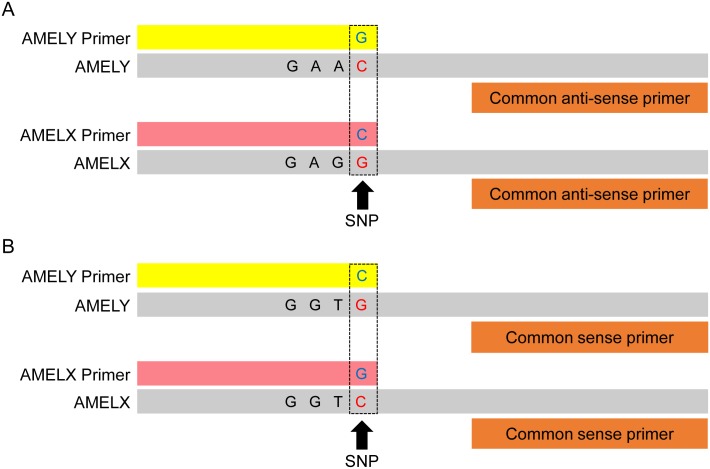
Schematic of the AMEL sense and antisense primer sets. The primer sequences are described in Table 1. A and B show sense and antisense primer sets, respectively. Yellow and pink boxes represent allele-specific primers. Orange boxes serve as common sense or antisense counter primers. Gray boxes indicate sense and antisense DNA templates. Blue letters show the 3ʹ-terminal bases in allele-specific primers, whereas red letters show C↔G transversion polymorphisms in sense and antisense DNA templates. Dotted boxes indicate base pairings at SNP sites between allele-specific primers and sense or antisense DNA templates.

As sense allele-specific primers, an AMELY primer with G at the 3ʹ-terminus and an AMELX primer with C at the 3ʹ-terminus were designed (Fig 1). A counter primer was designed using antisense strands of AMEL genes (Table 1). In addition, to avoid incorrect conclusions resulting from AMELY allelic dropout, the SRY gene was adopted. AMELY-, AMELX-, and SRY-specific primers amplified 60-, 56-, and 52-bp bands, respectively.

**Table 1:** Sense and antisense primer sets.

Name of primer set	Gene	Primer	Sequence	Final concentration (μM)	Amplicon size (bp)
Sense primer sets	Amelogenin Y	AMELY-Exon 2-A	IIII GGA TTT T***G***T TTG CCT GCC T***TG***	0.1	60
	Amelogenin X	AMELX-Exon 2-A	GGA TTT T***A***T TTG CCT GCC T***CC***	0.2	56
	Amelogenin	AMEL-Exon 2-A-R	IIIIIIIIII ACA GGC ATG GCA AAA GCT G	0.2	
	SRY	SRY-F	TCG GGT AAC ATT GGC TAC AAA GA	0.2	52
		SRY-R	GTT ATC GTA AAA AGG AGC ATC TAG GTA G	0.2	
Antisense primer sets	Amelogenin Y	AMELY-Exon 2-B	IIII TGG CAA AAG CTG CTC CCA ***C***	0.2	60
	Amelogenin X	AMELX-Exon 2-B	TGG CAA AAG CTG CTC CCA ***G***	0.2	56
	Amelogenin	AMEL-Exon 2-B-F	TCA AGA AAT GGG GAC CTG GAT T	0.2	
	SRY	SRY-F	TCG GGT AAC ATT GGC TAC AAA GA	0.2	52
		SRY-R	GTT ATC GTA AAA AGG AGC ATC TAG GTA G	0.2	

The letter ‘I’ stands for inosine. Underlined letters indicate 5ʹ-inosine flaps. SNP sites are indicated in bold and italics.

### Antisense primer sets

As antisense allele-specific primers, an AMELX primer with G at the 3ʹ-terminus and an AMELY primer with C at the 3ʹ-terminus were designed (Fig 1). A counter primer was designed using sense strands of AMEL genes (Table 1). In the antisense primer sets, AMELY-, AMELX-, and SRY-specific primers amplified 60-, 56-, and 52-bp bands, respectively.

### PCR conditions

Amplification was performed in a 10-μL reaction volume containing 1× Qiagen Multiplex PCR Master Mix (Qiagen, Hilden, Germany), 0.1–0.2 μM of each primer, and 1 μL of template DNA. Amplification was performed on a Takara PCR Thermal Cycler Fast (Takara, Otsu, Japan) using two different programs. Amplification of the sense primer sets was carried out using the following program: 95°C for 15 min of initial denaturation, followed by 38 cycles at 94°C for 30 s, annealing at 66°C for 90 s, and final extension at 72°C for 3 min. Amplification of the antisense primer sets was carried out using the following program: 95°C for 15 min; followed by 5 cycles at 94°C for 30 s and 64°C for 5 min; and then 33 cycles at 94°C for 30 s, 64°C for 90 s, and 72°C for 3 min.

### Polyacrylamide gel electrophoresis

All PCR products were mixed with 2 μL of 6× loading buffer (Takara) and electrophoresed together with a 10-bp ladder marker (Invitrogen, Carlsbad, CA, USA) on a 15% polyacrylamide gel (TEFCO, Tokyo, Japan) in 1× Tris borate EDTA buffer. The bands were visualized with SYBR Green I (Sigma, St. Louis, MO, USA) staining and a blue light transilluminator.

### STR typing

STR typing was performed using PowerPlex^®^ ESX17 Fast and PowerPlex^®^ Fusion systems (both Promega, Madison, WI, USA), in accordance with the manufacturer’s recommendations. Amplification products were analyzed with an Applied Biosystem^®^ 3130xl Genetic Analyzer (Thermo Fisher Scientific, Waltham, MA, USA) using a 3-kV, 5-s injection. Results were analyzed using 50 rfu as the peak amplitude threshold.

### Forensic application

**Fig 2:**
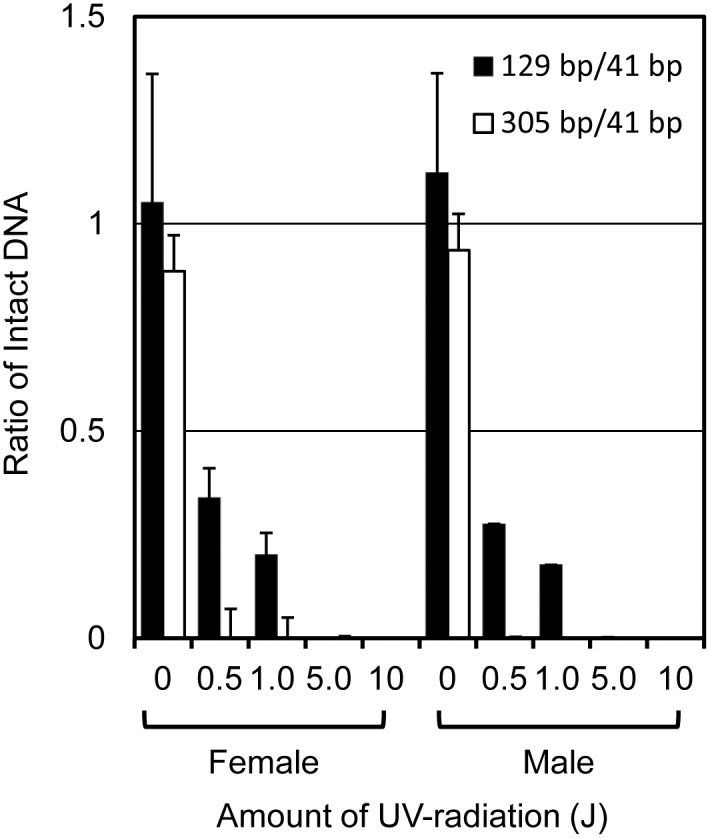
Assessment of the quality of mock forensic samples. Black and white columns show 129/41 bp and 305/41 bp Q-ratios, respectively. The Q-ratios correlate with the amount of UV radiation. Both female and male mock forensic samples with no UV exposure produced Q-ratios close to 1. Mock forensic samples subjected to high levels of UV irradiation yielded 129/41 bp and 305/41 bp ratios in the ranges of 0.00–0.38 and 0.00–0.038, respectively. Mean values ± SD are from triplicate assays.

DNA samples (1 ng) were artificially damaged by 0.2, 0.5, 1.0, 5.0, and 10 J ultraviolet (UV) irradiation and were used as mock forensic samples [52]. Q-ratios (129/41 and 305/41 bps) were calculated to evaluate the quality of the UV-damaged DNA template and assessed in triplicate using the KAPA Human Genomic DNA Quantification and QC Kit (Kapa Biosystems, Wilmington, MA, USA) and the Applied Biosystems StepOnePlus Real-Time PCR System (Thermo Fisher Scientific). The Q-ratios decreased in a UV irradiation-dependent manner (Fig 2). These DNA samples were analyzed using PowerPlex^®^ ESX17 Fast and PowerPlex^®^ Fusion systems, and our new method.

### Archeological application

To evaluate the effectiveness of our method for archeological samples, 14 late Jomon (about 3,500 years BP) DNA samples whose mitochondrial DNA had been successfully analyzed in previous studies [28, 29] were examined. The sex of these samples was determined unambiguously by morphological analysis of the skeletons [28]. Ancient DNA samples were analyzed using PowerPlex^®^ ESX17 Fast and PowerPlex^®^ Fusion systems, and our new method. We did not measure the concentration of this ancient DNA because it is difficult to determine this accurately for ancient samples using an absorption photometer given the scarcity and low quality of such DNA.

## Results

### Sex determination based on a novel PCR-APLP method

**Fig 3:**
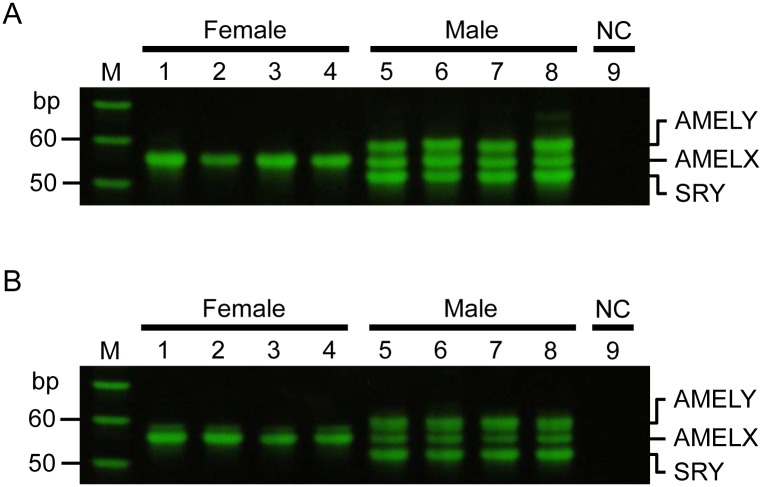
Sex determination based on a novel PCR-APLP method using 1 ng of template DNA. Lanes 1–4 correspond to female DNA, lanes 5–8 to male DNA, and lane 9 to the negative control. A and B show results of sex determination using the sense and antisense primer sets, respectively. M indicates the 10-bp ladder marker. The results were reproduced in three independent assays.

Both sense and antisense primer sets produced a single band of 56 bp (AMELX) in female samples, and male samples showed three bands of 60 bp (AMELY), 56 bp (AMELX), and 52 bp (SRY). The sex of the eight participants (four male, four female) was determined correctly (Fig 3).

### A sensitivity measurement for defining lower limits of quantitation

**Fig 4:**
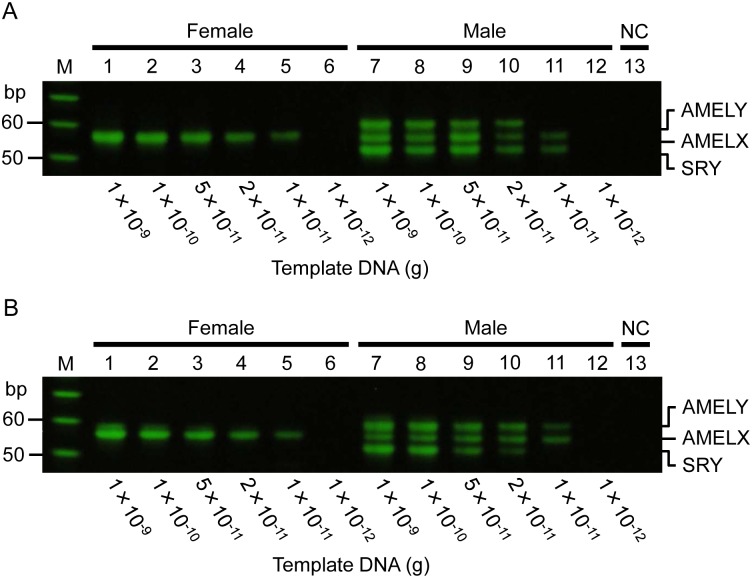
Sensitivity measurement by PCR-APLP using a dilution series of female and male DNA. Lanes 1–6 correspond to a serial dilution of female DNA, lanes 7–12 to a serial dilution of male DNA, and lane 13 to the negative control. A and B show results of sensitivity analysis using the sense and antisense primer sets, respectively. M indicates the 10-bp ladder marker. The results were reproduced in three independent assays.

To define the detection limit for accurate sex determination, male and female standards of known DNA concentrations were used. The minimum quantity required for accurate sex determination was 10 pg/μL for female and 20 pg/μL for male DNA (Fig 4).

### A comparative robustness evaluation of STR analysis and a novel PCR-APLP method

Results from the comparison of three methods, using UV-irradiated template DNA (1 ng), are shown in Table 2. Our method was more robust than the two conventional STR typing kits.

**Table 2:** Comparative robustness evaluation of STR analyses and a novel PCR-APLP method.

	APLP method		PowerPlex^®^ ESX17 Fast		PowerPlex^®^ Fusion	
Sex	Female	Male	Female	Male	Female	Male
Amount of UV irradiation (J)	1.0	1.0	0.5	0.2	1.0	0.5

**Fig 5:**
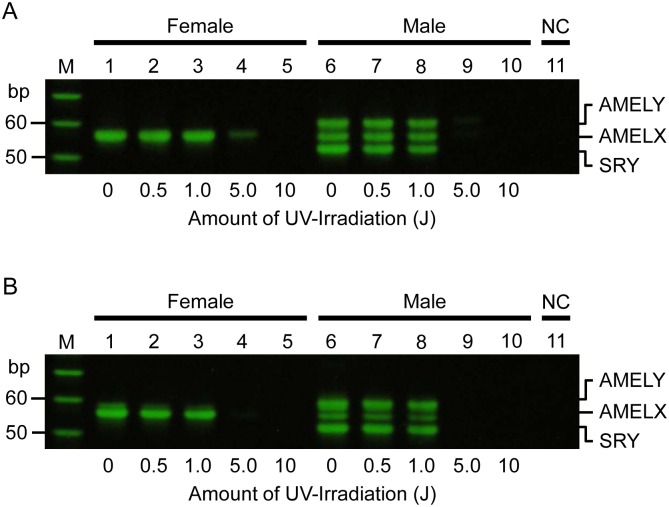
Robustness evaluation of a PCR-APLP method using UV-irradiated template DNA. A and B show the results of robustness analysis using the sense and antisense primer sets, respectively. Lanes 1–5 correspond to female DNA damaged by 0, 0.5, 1.0, 5.0, and 10 J UV irradiation; lanes 6–10 to male DNA damaged by 0, 0.5, 1.0, 5.0, and 10 J UV irradiation; and lane 11 to the negative control. Lanes 1 and 6 are female and male positive controls, respectively. M indicates the 10-bp ladder markers. The results were reproduced in three independent assays.

None of the PCR-APLP primer sets could detect a single band from 1 ng of female DNA or three bands from male DNA after UV irradiation of more than 1.0 J (Fig 5).

**Fig 6:**
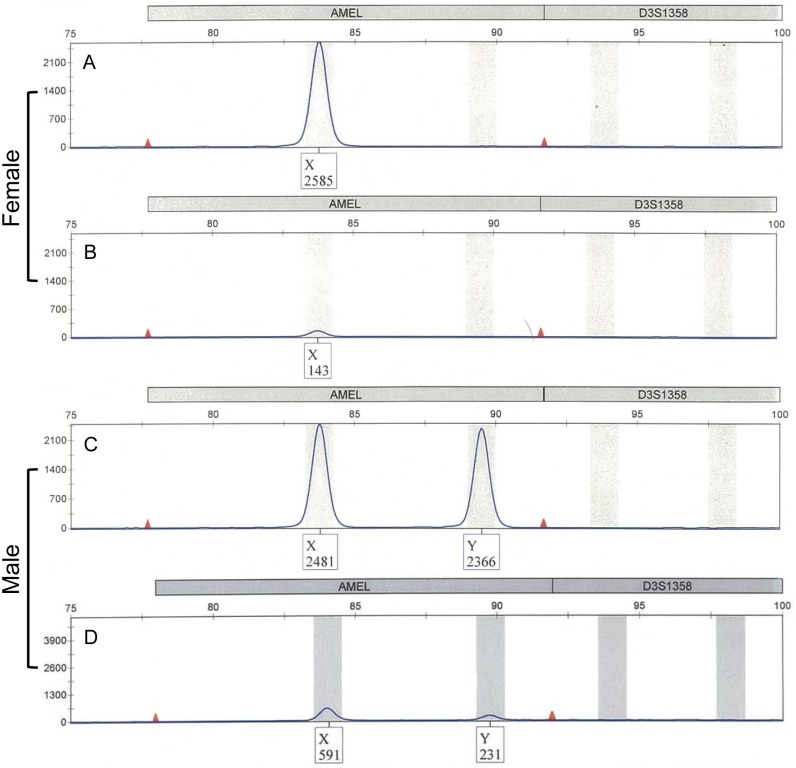
Sex determination by PowerPlex^®^ ESX17 Fast using UV-irradiated template DNA. A and C show female and male DNA with no UV irradiation, respectively. B and D show UV irradiation-exposed female (0.5 J) and male (0.2 J) DNA, respectively. The results were reproduced in three independent assays.

In the PowerPlex^®^ ESX17 Fast and the PowerPlex^®^ Fusion systems, female samples were identified by a single band of 89 bp (AMELX), whereas the presence of two bands of 89 bp (AMELX) and 95 bp (AMELY) indicates male genotypes. STR typing using PowerPlex^®^ ESX17 Fast was not possible with female DNA of 1 ng after UV irradiation of more than 0.5 J. For 1 ng of male DNA exposed to more than 0.2 J UV irradiation, neither AMELX nor AMELY was detected (Fig 6).

**Fig 7:**
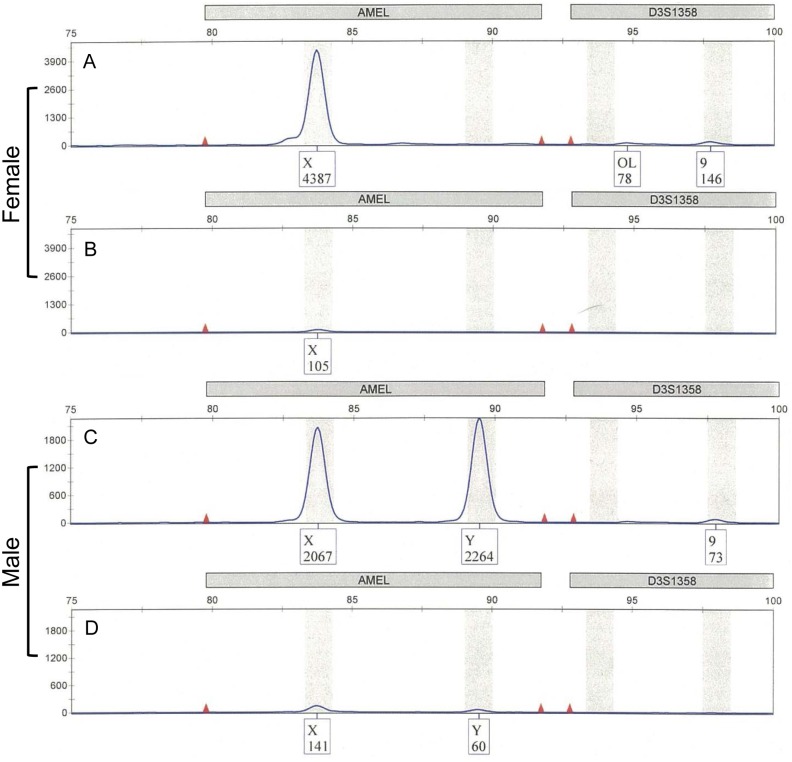
Sex determination by the PowerPlex^®^ Fusion system using UV-irradiated template DNA. A and C show female and male DNA with no UV irradiation, respectively. B and D show UV irradiation-exposed female (1.0 J) and male (0.5 J) DNA, respectively. The results were reproduced in three independent assays.

STR typing using the PowerPlex^®^ Fusion system was not possible with 1 ng of female DNA after UV irradiation of more than 1.0 J. After exposure to more than 0.5 J UV irradiation, two peaks were no longer observed in male samples (Fig 7).

### Archeological application

**Fig 8:**
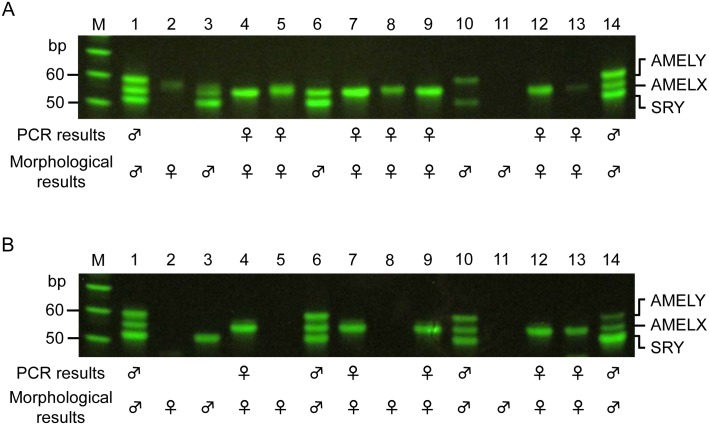
DNA sexing of Jomon samples by our method. A and B show the results of sex determination using the sense and antisense primer sets, respectively. M indicates the 10-bp ladder marker. The results were reproduced in three independent assays. Typical results of bidirectional analysis are shown because amounts of template DNA were not constant among ancient samples.

The sense primer sets and antisense primer sets could successfully determine the sex of 9 out of 14 samples (lanes 1, 4, 5, 7, 8, 9, 12, 13, and 14 in Fig 8A) and 9 out of 14 samples (lanes 1, 4, 6, 7, 9, 10, 12, 13, and 14 in Fig 8B), respectively. We could determine the sex of 11 out of 14 individuals by using both of the primer sets. The results of DNA sexing corresponded exactly to those obtained by morphological analysis.

Owing to the small sample volume, we could not perform STR analyses on the ancient sample electrophoresed in lane 1 in Fig 8A and 8B. Other samples were analyzed twice by using the PowerPlex^®^ ESX17 Fast and PowerPlex^®^ Fusion systems. Reproducible successful results were obtained from the samples electrophoresed in lanes 7 and 9 in Fig 8A and 8B for PowerPlex^®^ ESX17 Fast, and the samples electrophoresed in lanes 7, 9, and 12 in Fig 8A and 8B for the PowerPlex^®^ Fusion system (data not shown).

## Discussion

In this study, we developed a new sex determination method, the sense–antisense AMEL PCR-APLP assay with SRY analysis. All DNA samples from the eight participants (four male, four female) were evaluated correctly by our method. Our sex determination test had detection limits of 10 pg for female DNA and 20 pg for male DNA, which corresponds to the theoretical DNA copy number of a few copy numbers. Even the PowerPlex^®^ ESX17 Fast and the PowerPlex^®^ Fusion systems, which are well known to be more tolerant of degraded DNA than other STR kits, require 250–500 pg of DNA template. These results showed that our method had higher sensitivity than other STR kits. Moreover, allelic dropout occurred for samples with less than 125 pg of template DNA in the PowerPlex^®^ Fusion system [53]. In the PowerPlex^®^ Fusion system, because of their length, the AMEL genes seem inherently more tolerant to allelic dropout than other STR loci; however, when allelic dropout occurs for some STR loci, the possibility that a male is falsely genotyped as a female due to allelic dropout of the Y chromosome cannot be ruled out. Because forensic laboratories often have to deal with low-quantity DNA samples for criminal investigations as well as ancient remains that tend to contain small quantities of DNA, our new highly sensitive method is extremely promising.

Forensic and ancient DNA samples are often highly fragmented. We designed primers that amplify fragments within the range of 52–60 bp because shorter PCR products are desirable [14]. Compared with two commercial STR analysis kits, our new method was more robust for analyzing artificially fragmented DNA samples. The AMEL fragments amplified with our method (AMELX: 56 bp, AMELY: 60 bp) were shorter than those amplified with the PowerPlex^®^ ESX17 Fast and the PowerPlex^®^ Fusion systems (AMELX: 89 bp, AMELY: 95 bp). Because our amplicons are <60 bp in size, smaller than amplicons produced by any other STR analysis kit, our method is very useful for analyzing heavily fragmented samples. When either AMELX or AMELY is not generated due to allelic dropout, our method gives a result of no identification; however, no amplification of AMELY due to allelic dropout, which indicates a false conclusion of males as females, can occur in STR analysis when only AMELX is detected in samples. Our novel method could detect Y chromosomes even in male DNA subjected to 1.0 J of UV irradiation, and it was robust with highly fragmented DNA samples. In 1 ng of UV-damaged male DNA sample (0.5 J), although AMELX was detected, AMELY was not detected by STR analysis using the PowerPlex^®^ ESX17 Fast system. As with PowerPlex^®^ ESX17 Fast, the PowerPlex^®^ Fusion system could detect only AMELX using 1 ng of male DNA UV-damaged by 1.0 J of irradiation. These results show the difficulty of STR DNA typing from degraded samples and indicate the importance of using additional loci to complement allelic dropout. Thus, our method adopted the SRY gene to complement AMEL allelic dropout.

It is well known that male samples are sometimes falsely determined as female owing to Y chromosome deletions. Several reports have indicated that there are ethnic differences in the frequency of AMELY deletion. The frequency of AMELY deletion was high in male samples coming from India, Nepal, and Sri Lanka [4, 34, 36, 39–42] and, conversely, low in some populations, including Chinese, Italian, Australian, and Spanish ones [35, 38, 41, 42, 44–46, 49, 50]. Failure to amplify AMELY could lead to erroneous results in forensic practice and archeological work; therefore, it should be taken into careful consideration even in such populations. The existence of male AMELY deletions suggests the need for simultaneous analysis of multiple loci to avoid false identification and, in such deletion cases, when AMELY is not amplified, the SRY gene would be.

In this study, we analyzed 14 Jomon samples by our new method, and this method was proved more effective for archeological samples than commercial STR kits. Besides testing the robustness of our method, the purpose of this analysis was to evaluate the influence of deamination for both sense and antisense strands of AMEL genes because the archeological remains used in this assay came from an area with a cold climate, northern Hokkaido [28, 29]. Several previous studies have indicated that types of damage present in archeological remains are associated with specific geographical regions or climatic conditions [21, 23, 54, 55], with cold locations contributing to high levels of hydrolytic deaminated DNA. The main *postmortem* DNA modifications are type 2 transitions (C→T and G→A) [22–24]. In our method, because a sense AMELY primer with G at the 3ʹ-terminus detects C in the AMELY genes, when C is modified to U by deamination, the sense AMELY primer will not give an AMELY amplification product. Therefore, if a C to U conversion occurred in a male sample, the AMELY gene could not be detected by our primer sets. In this case, because AMELX will not cause allelic dropout owing to the deamination process, we are able to deal with female samples that are suspected to be deaminated. Deaminated male samples will result in allelic dropout of AMELY, but we can reach the conclusion that an analyzed sample originates from a male by the presence of AMELX and SRY bands at 56 bp and 52 bp. Conversely, because an antisense AMELX primer with G at the 3ʹ-terminus detects C in the AMELX genes, when C is modified to U by deamination, the antisense AMELX primer will not give any AMELX amplification products. Therefore, if the conversion of C to U occurred in a female sample, amplification failure of AMELX hinders the sex determination; however, we would not have incorrect sex determination results owing to allelic dropout. In forensic samples, avoiding erroneous results is more important than having no results because false accusations can ruin lives.

In the sense primer sets, AMELY dropout, probably resulting from deamination, was detected in two out of six morphologically male samples (lanes 3 and 6 in Fig 8A). In the antisense primer sets, AMELX dropout, also presumably resulting from deamination, was found in two out of eight morphologically female samples (lanes 5 and 8 in Fig 8B) and in one out of six morphologically male samples (lane 3 in Fig 8B). From both sense and antisense analyses, we can assume that these four samples are most likely influenced by deamination. We do not have false sex determination results in both primer sets, and this “bidirectional analysis” method allows a much higher identification rate than analysis based on PCR amplification of only one strand of the target DNA. These results indicate the importance of analyzing samples from both sense and antisense strands in highly degenerated DNA samples. This bidirectional analysis of degenerated DNA samples would improve the reliability of various SNP analysis methods, such as SNaP shot and single-strand conformation polymorphism, as well as APLP.

In the present study, we showed how the inosine-flapped APLP bidirectional SNP analysis of amelogenin and SRY genes can be efficiently applied to sex determination for highly fragmented and degenerated DNA samples. Primers with a 5′-flap have been used to improve the efficiency of the PCR reaction [56–59] and to enhance the quality of sequencing [60]. Such primers have also been reported to increase the fluorescent signal of real-time PCR [56, 57]. In addition, inosine-flapped APLP primers improve the competitiveness of allele-specific primers to the template DNA, resulting in enhanced reliability of the SNP analysis [33]. The thermodynamics of the primers with inosine flaps has been proven to be less influenced by the sequence of PCR templates than the thermodynamics of the primers with 5′-flaps containing ordinary bases [33]. Furthermore, we reported that our inosine-flapped APLP method in the multiplex SNP analysis showed markedly higher sensitivity than that reported in various studies [61]. These features of 5′-flap primers are likely to have contributed to the high sensitivity observed in our sex determination method.

Unfortunately, this study employed only two commercial standard DNA, eight modern DNA, and 14 ancient DNA samples. This number of DNA samples may not be sufficient in the field of forensics. As mentioned earlier, there are various problems with the dropout of alleles used to determine sex in different geographical populations. However, the problems caused by genetic polymorphism are inevitable in sex determination by using DNA analyses, even if the sample size is enlarged. In the near future, however, we will investigate the possibility of the dropout of alleles used to determine sex in our method by using a greater number of DNA samples.

In conclusion, we established a new PCR-APLP method for sex determination by analyzing SNPs in AMEL exon 2 from both sense and antisense strands in conjunction with SRY testing, resulting in a higher identification rate than analysis based on PCR amplification of only one strand of the target DNA.
